# A single oral administration of flavanols enhances short-term memory in mice along with increased brain-derived neurotrophic factor

**DOI:** 10.1515/med-2025-1270

**Published:** 2025-10-08

**Authors:** Yasuyuki Fujii, Yuki Yamato, Minoru Shibata, Kazunari Takano, Sota Tsuchibuchi, Shu Taira, Vittorio Calabrese, Naomi Osakabe

**Affiliations:** SIT Research Laboratories, Shibaura Institute of Technology, 307 Fukasaku, Minumaku, Saitama, 337-8570, Japan; Functional Control Systems, Graduate School of Engineering and Science, Shibaura Institute of Technology, Saitama, 337-8570, Japan; Faculty of Food and Agricultural Sciences, Fukushima University, Fukushima, 960-1296, Japan; Department of Biomedical and Biotechnological Sciences, University of Catania, Catania, 95131, Italy

**Keywords:** flavanol, novel object test, short-term memory, CREB, BDNF

## Abstract

**Background:**

Flavanols (FLs), a group of polyphenols abundant in cocoa and red wine, have been associated with improved cognitive function, particularly hippocampus-dependent memory in older adults. However, their poor bioavailability has limited understanding of the mechanisms underlying their effects on the brain. This study aimed to investigate the cognitive and molecular responses following acute FLs administration in mice.

**Methods:**

Male C57BL/6J mice were orally administered FLs (25 mg/kg) or distilled water (DW) and subjected to a novel object recognition test. In one experiment, FLs were administered before training, in another, after training. Exploratory behavior and the discrimination index (DI) were analyzed. Hippocampal tissues were collected at 15 min to 4 h post-administration to assess levels of brain-derived neurotrophic factor (BDNF) and phosphorylated extracellular signal-regulated kinase, cAMP response element-binding protein (CREB), and tyrosine kinase receptor type 2 by western blotting.

**Results:**

Mice treated with FLs before training exhibited significantly longer exploration of the novel object and higher DI, whereas no enhancement was observed when FLs were administered after training. CREB phosphorylation increased at 30 min post-administration, and BDNF levels were elevated at 2 and 4 h.

**Conclusion:**

These findings suggest that FLs enhance short-term memory via hippocampal CREB activation and BDNF upregulation. Despite low systemic absorption, the rapid effects observed may involve sensory signaling pathways, potentially triggered by the astringent properties of FLs. This study provides mechanistic insight into the cognitive benefits of dietary FLs.

## 1 Introduction

**Figure 1:**
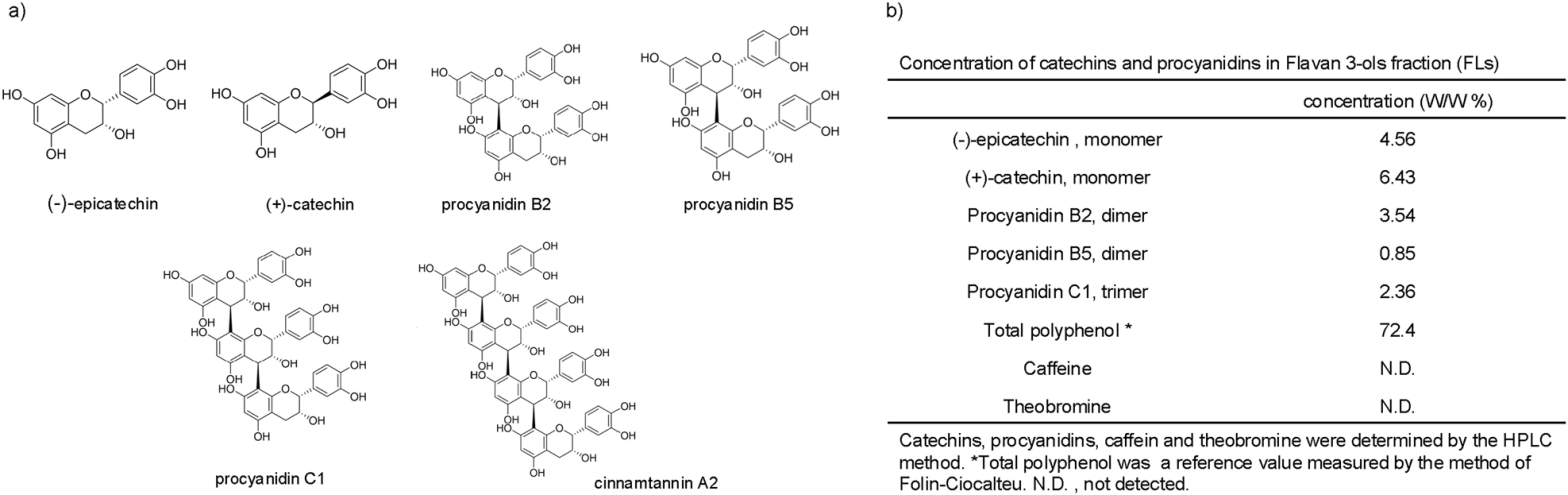
Overview of the flavan-3-ols fraction used. (a) The structural formula of flavan 3-ols top row (from left to right): (-)-epicatechin, (+)-catechin, procyanidin B2, procyanidin B5, bottom row: procyanidin C1, cinnamtannin A2 and (b) composition of flavan-3-ols fraction.

Flavanols (FLs) are a group of polyphenols comprising (−)-epicatechin and its oligomeric procyanidins, procyanidin B2(dimer), procyanidin C1(trimer), and cinnamtannin A2(tetramer Figure 1a). A strong astringent taste characterizes them and is present in high concentrations in cocoa, red wine, and berries [1]. Previous systematic reviews showed that FLs may play a role in maintaining or improving cognitive function [2,3]. Furthermore, a large-scale intervention study reported that 1 year of FL intake restored hippocampal-dependent memory in elderly participants with a low habitual dietary quality [4]. Furthermore, several intervention studies have reported significant changes in brain function soon after ingestion of FLs-rich food [5], e.g., increasing mental tracking capacity [6,7], mood [8], attention [9], restored working memory performance [10 11,12], and enhancement of the peripheral circulation [13,14]. However, the poor absorption of FLs from the digestive tract and their limited distribution in the blood or brain [1,15] remain unclear in their precise mechanism of action. A preceding study demonstrated that repeated intragastric administration of cinnamtannin A2, the most bioactive epicatechin derivative in FL, enhanced spatial memory and promoted neurogenesis in mice hippocampal dentate gyrus [16]. Despite growing evidence from long-term or short-term human intervention studies showing cognitive benefits of FLs, the underlying molecular mechanisms remain poorly understood. This is primarily due to the poor absorption of FLs and their restricted distribution to the brain. Therefore, the present study aimed to use a mouse model to directly assess the short-term responses in the hippocampus of memory-related molecules after acute FL administration, thereby providing insights into how and when dietary FLs may enhance cognitive function.

In the present study, a novel object test (NOT) was employed to investigate the impact of a single oral administration of FLs on memory in mice. The present study also examined the effect of the timing of FL administration on brain function. Additionally, the time course of brain-derived neurotrophic factor (BDNF) production in the hippocampus, extracellular signal-regulated kinase (ERK), transcription factor cAMP response element binding protein (CREB), and phosphorylation of neurotrophic tyrosine kinase receptor type 2 (TrkB) following FL administration was observed.

## 2 Materials and methods

### 2.1 Materials

The composition of FLs derived from cocoa used in the experiment is shown in Figure 1b.

In addition, xanthine derivatives (caffeine, theobromine; Sigma-Aldrich, Japan) were below the detection limit. Catechins, procyanidins, and xanthine derivatives were measured according to the method of Natsume et al. [17]. To ensure complete dispersion of the dosing solution, the preparation process included 1 min of sonication followed by 10 s of vortexing, repeated twice.

### 2.2 Animals

Ten weeks old male C57BL/6J mice were obtained from CLEA Japan, Inc. (Tokyo, Japan). Male mice were used to minimize variability caused by hormonal cycles, which are known to affect cognitive function. During 2 weeks acclimation period, the animals were carefully handled to reduce anxiety behaviors. Mice were housed at room temperature (24–26°C) under a 12 h light/dark cycle (light cycle: 7:00–19:00, dark cycle: 19:00–7:00) with free access to water and food. The solid diet (MF) for laboratory animals was obtained from Oriental Yeast Co., Ltd (Tokyo, Japan). The study was conducted in accordance with the Code of Ethics in compliance with the Declaration of Helsinki, and the protocol was approved by the Animal Experimentation Committee of Shibaura Institute of Technology (approval number: AEA 23006). In addition, all animals were humanely raised according to the guidelines of this period according to ARRIVE guidelines.

### 2.3 Novel object recognition test

**Figure 2:**
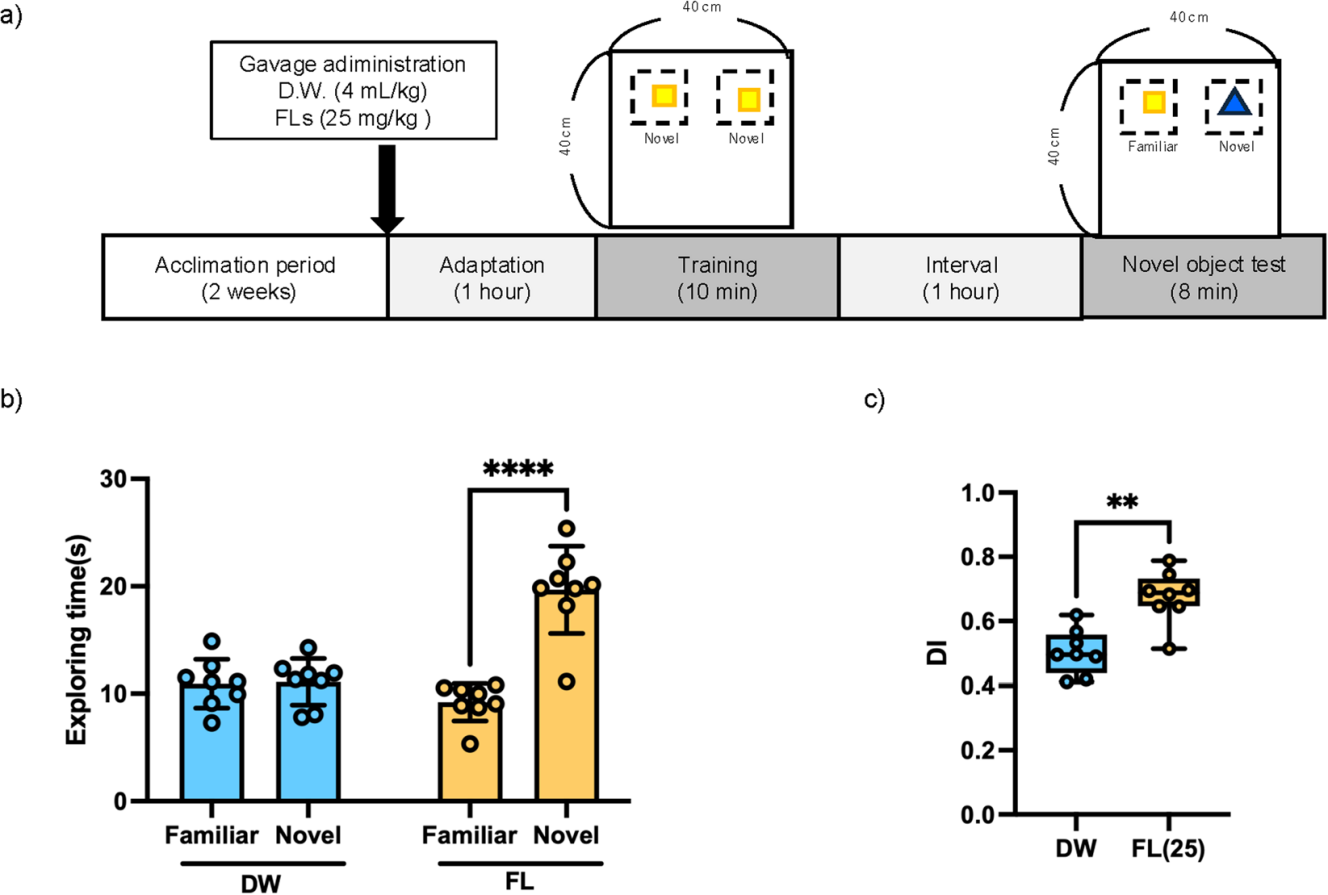
Results of the novel object recognition test (NOT) following the administration of the test reagents before training phase. (a) Study design of NOT, (b) total exploration time in NOT, (c) DI data are expressed as mean ± standard deviation. Two-way ANOVA was performed for total exploration time, followed by Bonferroni’s multiple comparisons test. For DI, the non-parametric Mann–Whitney test was conducted. ****p < 0.0001 vs familiar; **p < 0.01 vs DW.

**Figure 3:**
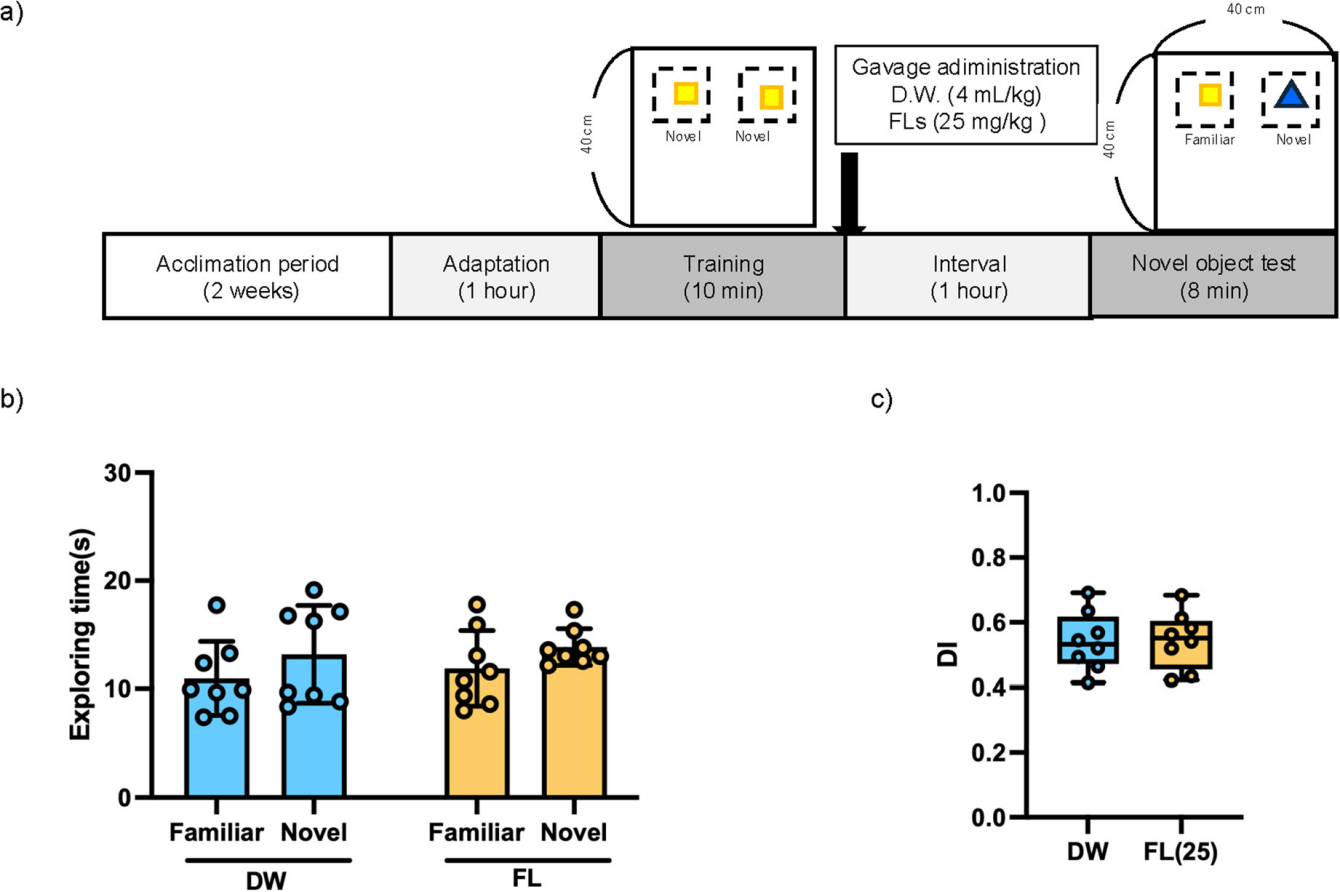
Results of the novel object recognition test (NOT) following the administration of the test reagents after the training phase. (a) Study design of NOT, (b) total exploration time in NOT, (c) DI data are expressed as mean ± standard deviation. Two-way ANOVA was performed for total exploration time, followed by Bonferroni’s multiple comparisons test. For DI, the non-parametric Mann–Whitney test was conducted.

The novel object recognition test was performed according to the method of Leger et al. [18]. After 2 weeks of acclimation, mice were divided into two groups, one treated with distilled water (DW) and the other with FL 25 mg/kg (*n* = 8 each). Our previous studies have shown that a single oral dose of 10–50 mg/kg of FL stimulates the brain and induces a stress response, so we selected a moderate dose of 25 mg/kg in this study. In the first experiment, mice given DW or FL by gavage administration were placed in the center of a transparent acrylic arena (40 × 40 × 40 cm) and allowed to adapt for 1 h, as shown in Figure 2a. Two identical objects were placed in opposite quadrants of the arena, and the mice were allowed to explore them freely for 10 min. After 1 h, one of the objects was replaced with a new object with a different shape and material, and the mice were allowed to explore the arena for 10 min to perform the novel object recognition test. In the second experiment, as shown in Figure 3a, mice were allowed to freely explore two identical objects for 10 min, after which they were gavage administered DW or FLs, and 1 h later one of the objects was replaced with a novel object with a different shape and material and allowed to explore for 10 min. The decision to explore or not to explore was analyzed from the data using Premier Pro (Adobe Inc., California, USA) as described above [19]. To assess working memory, the discrimination index (DI) was calculated as the time spent exploring the novel object divided by the total exploration time, as previously described in Leger et al. [18] as follows: [(time spent exploring the novel object) − (time spent exploring the familiar object)]/[(time spent exploring the novel object) + (time spent exploring the familiar object)].

### 2.4 Western blotting

To detect phosphorylation of ERK or CREB immediately after FLs administration, the hippocampi were excised 15 or 30 min after intragastric administration of DW or 25 mg/kg FLs to mice (*n* = 8 each), and stored at −80°C. To observe BDNF production and TrKB phosphorylation, hippocampus was excised from similarly treated mice 1, 2, and 4 h later and stored frozen. Tissues were homogenized in a microtube with lysis buffer (CelLyticTM MT Cell Lysis Reagent; Sigma-Aldrich, Japan) containing a protease inhibitor (Sigma-Aldrich, Japan) and 0.2% w/v SDS. Protein concentration was measured using the Bradford method. Protein (20 µg) was separated by SDS-PAGE using a 10–20% Bis-Tris gel and transferred onto a polyvinylidene difluoride membrane (Life Technologies, CA, USA).

To quantify total protein, Ponceau S staining was performed. The membrane was immersed in Ponceau S staining solution (P7170-1L, Sigma-Aldrich, St. Louis, MO, USA) and gently shaken for 5 min. Next, the membrane was immersed in 1% acetic acid solution and gently shaken until a reduction in background and clear protein staining were observed. The processing time was approximately 2 min, but adjustments were made based on visual confirmation of staining. Images were captured using a scanner (EPSON GT-F740, Seiko Epson Corporation, Suwa, Nagano, Japan). Subsequently, the membrane was fully immersed in 0.1 M NaOH solution and gently shaken for destaining. The processing time was approximately 30–40 s. Finally, the membrane was washed by gently shaking it in Milli-Q water for 2 min.

For 1 h, the membrane was blocked with a membrane-blocking reagent (GE Healthcare, Buckinghamshire, UK). After blocking, the membrane was incubated with a rabbit polyclonal primary anti-β-actin (1:1,000; sc-47778, Santa Cruz Biotechnology, Dallas, USA), anti-ERK (MK1; sc-135900, Santa Cruz Biotechnology), anti-phosphoERK (12D4; sc-81492, Santa Cruz Biotechnology), anti-CREB (48H2, 1:1,000; #9197, Cell Signaling, MA, USA), anti-phospho CREB (Ser133, 1:1,000; #9198, Cell Signaling), anti-BDNF (EPR1292, 1:1,000, ab108319; Abcam, Cambridge, UK), anti-TrkB (80E3; #4603, Cell Signaling), and anti-phospho TrkA (Tyr490)/TrkB (Tyr516) (1:1,000; #4619, Cell Signaling). After the primary antibody reaction, the membrane was incubated with appropriate horseradish peroxidase-conjugated secondary antibodies (1:10,000, anti-rabbit IgG, HRP-linked whole Ab sheep; NA931, GE Healthcare, Buckinghamshire, UK) or anti-rabbit IgG, HRP-linked whole Ab donkey (NA934; GE Healthcare) for 1 h. Chemiluminescence was used to detect immunoreactivity using the ECL Select Western Blotting Reagent (GE Healthcare, Buckinghamshire, UK). Fluorescence band images were analyzed using Just TLC (SWEDAY, Larkgatan, Sweden) analysis software.

The ratio of Ucp-1 to α-tubulin for each animal was calculated.

### 2.5 Statistical analysis

The sample size was determined from the results of a preliminary experiment using a power test with a significance level of 0.05 and a power of 0.9. All quantitative assessments were carried out in a blind manner. No data were excluded from the analysis. All data were expressed as mean ± standard deviation. The data were analyzed using GraphPad Prism 10.2.3 (San Diego, CA, USA). The normal distribution of the sample was tested using the Shapiro–Wilk test. Two-way ANOVA followed by Tukey’s multiple comparisons test as a *post hoc* test was used for multiple comparisons. For DI, statistical analysis conducted by Mann–Whitney test. The significance levels were defined at *p < 0.05, **p < 0.01, ***p < 0.001, ****p < 0.0001.


**Ethical approval:** The study was conducted in accordance with the Code of Ethics in compliance with the Declaration of Helsinki, and the protocol was approved by the Animal Experimentation Committee of Shibaura Institute of Technology (approval number: AEA 23006).

## 3 Results

### 3.1 Results of novel object recognition test

Figure 2b shows the total exploration time for two objects when mice were administered SW or FL before the training period. There was no difference in the exploration time of the mice to the existing object or the new object in the DW group. In the FL group, the exploration time to the new object was significantly longer than that to the existing object. The DI value shown in Figure 2c was significantly higher in the FL group than in the DW group.

Figure 3b shows the total exploration time for two objects when mice were administered SW or FL after the training period. There was no difference in the exploration time of mice in both the DW and FL groups for the existing or novel objects. The DI values shown in Figure 3c also showed no difference between the DW and FL groups.

### 3.2 Change of BDNF and phosphorylated ERK, CREB, and TrkB after gavage administration of FL

**Figure 4:**
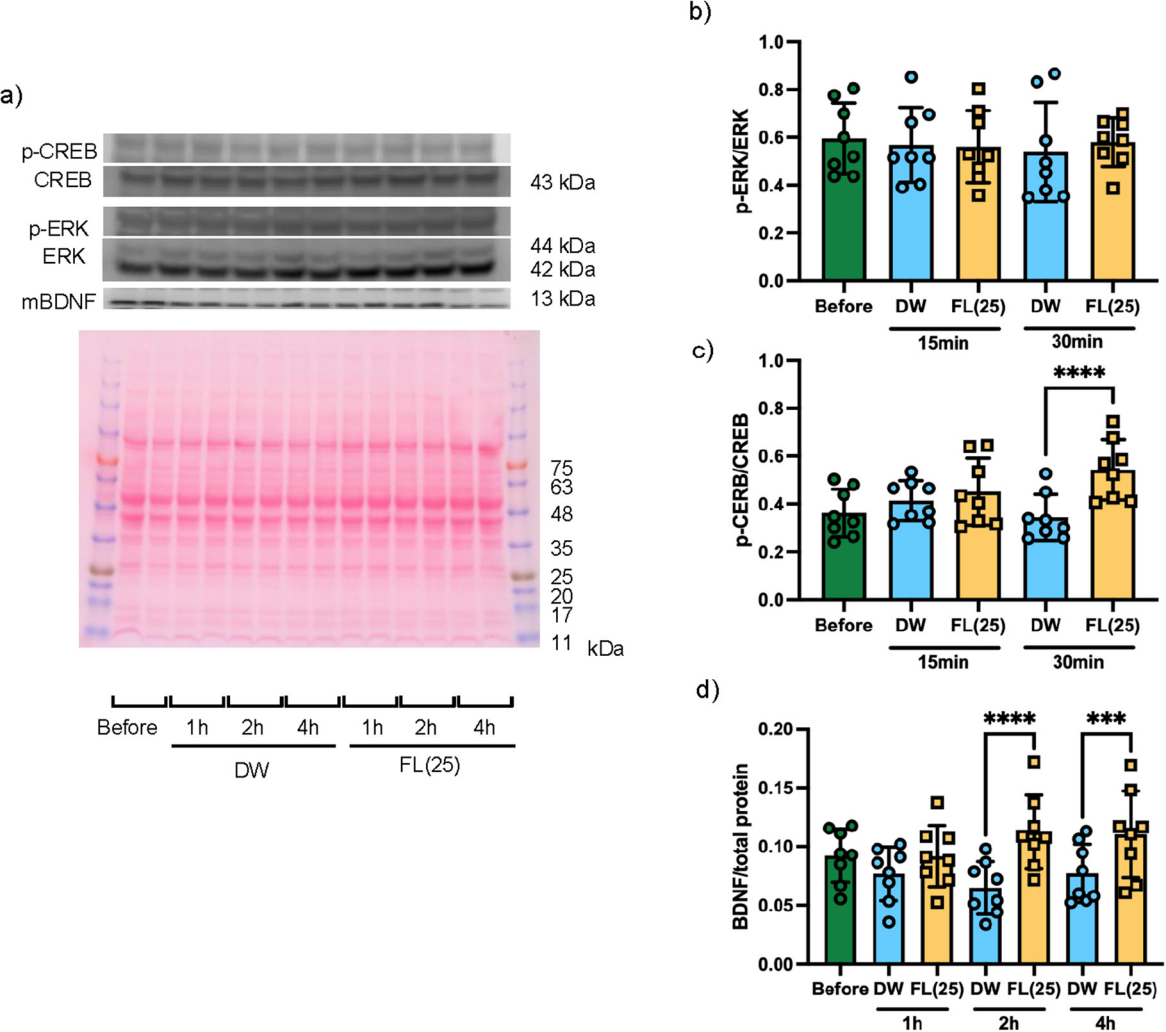
Quantitative results of memory-related protein expression following a single oral administration of flavan-3-ols. (a) Representative blot of protein expressions of ERK (42, 44 kDa), CREB (43 kDa), BDNF (13 kDa), and TrkB (90, 140 kDa) in the hippocampus. (b) Total protein profile using Ponceau staining. (b)–(d) Quantitative analysis of the optical density ratio of p-ERK/ERK, p-CREB/CREB, BDNF/total protein, and p-TrkB/TrkB. Data are expressed as mean ± standard deviation. Two-way ANOVA was performed for the ratio of memory-related protein expression, followed by Tukey’s multiple comparisons test. *p < 0.05, **p < 0.01, ***p < 0.001, ****p < 0.0001 vs DW.

The phosphorylated ERK or CREB levels 15 or 30 min after intragastric administration of DW or 25 mg/kg FL to mice are shown in Figure 4a or b. No difference was observed between the experimental groups in the ratio of phosphorylated ERK and ERK at any observation time. At 30 min after administration, the ratio of phosphorylated CREB and CREB was significantly increased in the FL group compared to the DW group. The levels of BDNF expression or phosphorylated TrkB 1, 2, or 4 h after intragastric administration of DW or 25 mg/kg FL in mice are shown in Figure 4c or d. At 2 and 4 h after administration, the expression of BDNF was significantly increased in the FL group compared with the DW group. No differences were observed in the ratios of phosphorylated TrkB and TrkB between the experimental groups at any observation time.

## 4 Discussion

As memory deterioration occurs with advancing age, resulting in mild cognitive impairment and dementia, the preservation of memory becomes a pivotal aspect in developing effective human ageing strategies [20]. A primary focus of research in this field has been on the development and implementation of prevention strategies, given the dearth of effective treatment options [21]. For instance, physical activity has been demonstrated to enhance cerebral blood flow, stimulate the production of neurotrophic factors, such as BDNF, augment brain volume, and preserve cognitive function [22,23]. Consequently, at present, physical exercise is recommended as a preventative measure for older adults with mild cognitive impairment [24].

In the context of the development of preventative methodologies for memory impairment, a significant number of techniques employed for the study of memory, including single-electrode cortical recordings and depth recordings, present considerable challenges in terms of their application to healthy subjects [25,26]. Therefore, it is imperative to utilize model systems employing experimental animals. To date, many experimental methods have been developed to evaluate the spatial memory of rodents, such as the Y-maze test, Morris water maze test, open field habituation test, and gait function test, and to evaluate reference/working memory, such as the 8-arm radial maze test, radial arm water maze, and novel object recognition test [27]. The novel object test used in this study is a method for assessing reference/working memory that does not require external motivation, reward, or punishment, but relies solely on the innate exploration of rodents [18].

Moreover, it has been documented that the ingestion of FLs, the focus of the present study, exerts a substantial influence on cognitive function. In a large-scale intake study conducted in recent years, when 500 mg of FLs was taken per day for an average of 3.6 years, an increase in list learning and recall episodic memory, called the Modified Rey Auditory Verbal Learning Test, was confirmed in subjects with a low quality of life [4]. In addition to chronic administration, a comprehensive review has demonstrated that a single oral dose of FL, in addition to chronic administration, can enhance cognitive function, including attention, mood, and memory, with these effects becoming apparent within a time frame of 30 min to an hour following ingestion [3].

The present study utilized experimental animals and employed a specific FL administration protocol, namely FL administration prior to training, followed by the performance of the NOT test 2 h later. This experimental design resulted in a significant increase in exploration time for a novel object. Conversely, no such effect was observed when the NOT test was conducted after training, i.e., 1 h after FL administration. These findings pointed out that the timing of FL administration may have a significant effect on changes in brain function. Therefore, an investigation was conducted into the time course of memory-related proteins in the hippocampus. The results of this investigation revealed that CREB was significantly phosphorylated 30 min after FL administration, and that BDNF expression was significantly increased 2–4 h after administration (Figure 4).

Memory is defined as the ability to store, retain, and retrieve information, and the creation and use of memory consist of three steps: encoding, which registers and consolidates received information; storage; and retrieval [28]. In this memory process, CREB phosphorylation following hippocampal G protein-coupled receptors (GPCRs) activation plays a crucial role. D1-type dopamine and β-adrenergic receptors are known to be important GPCRs in the hippocampus and are known to regulate memory and synaptic plasticity through CREB-dependent signaling [29]. These receptors are known to be activated by dopamine and noradrenaline, which are secreted from the locus coeruleus or ventral tegmental area in response to new stimuli [30]. The present study observed CREB phosphorylation 30 min after the administration of FL, hypothesizing that this was due to the activation of the GPCRs involved. Phosphorylated CREB has been shown to bind to specific sequences in the BDNF promoter, thereby regulating its transcription [30], thus positioning CREB as a pivotal regulator of neurotrophin responses. On the other hand, FL is hardly absorbed and a part of it is decomposed by intestinal bacteria to produce characteristic metabolites such as phenyl-γ-valerolactones (gVLM) [1]. Many researchers therefore believe that the various beneficial effects of flavonols may be mediated by these metabolites [31]. The two peaks of gVLM plasma concentrations (*C*_max_) of 260 and 88 nmol/L gVLM were observed at 1.8 and 5.3 h (*T*_max_) after FL ingestion [31]. On the other hand, because changes in brain function were observed 30 min to 1 h after a single dose of FL [3], it is believed that changes in the central nervous system occur well before the peak of gVLM blood concentration. Furthermore, our results confirmed strong phosphorylation of CREB in the hippocampus 30 min after administration. The present findings on the time course of a series of memory involvement proteins support those of a prior intervention study that examined the effects of a single dose of FL on brain function enhancement [3]. Therefore, it is reasonable to hypothesize that factors other than metabolites produced in the gut contribute to the alterations in brain function in FL.

ProBDNF and mBDNF have been demonstrated to induce synaptic long-term depression (LTD) and long-term potentiation (LTP) through their interaction with p75(NTR) and TrkB receptors, respectively, thereby promoting memory retention and synaptic plasticity [32]. The administration of FL was suggested that the activation of memory-related GPCRs, which subsequently leads to the phosphorylation of CREB and the production of BDNF, thus amplifying LTP and LTD, consequently, facilitating memory enhancement. In addition, our previous work demonstrated that repeated intake of cinnamtannin A2, a tetrameric form of (−)-epicatechin contained in FL, promotes neurogenesis in the hippocampal dentate gyrus and enhances cognitive function [15]. These findings suggest that the enhancement of BDNF synthesis observed in the present study maybe attributable to the action of cinnamtannin A2. This mechanism is likely to contribute similarly to the cognitive improvements seen in human intervention trials.

**Figure 5:**
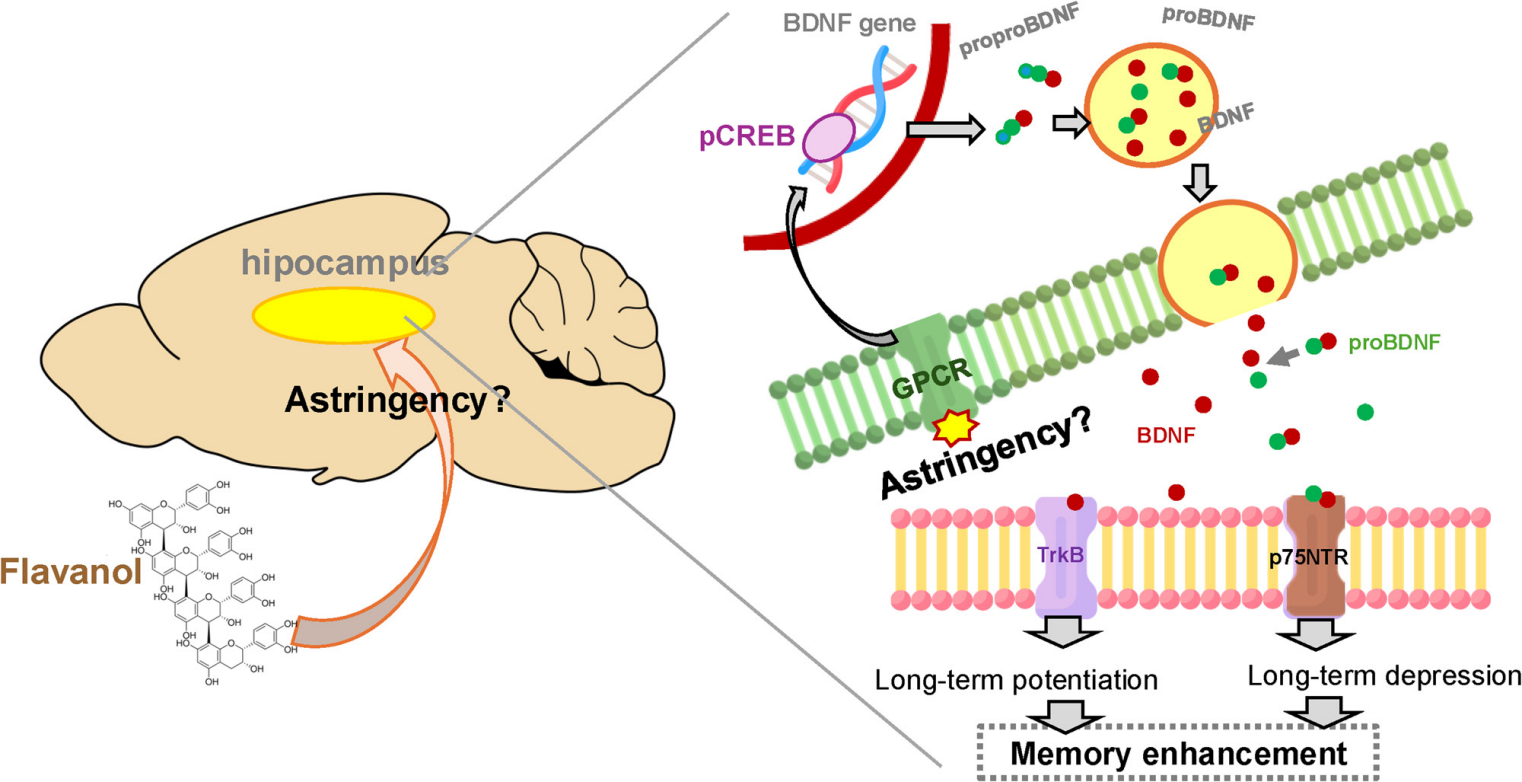
Hypothetical mechanism by which flavan-3-ols enhance hippocampus-dependent memory. We propose that astringency-induced activation of hippocampal GPCRs triggers CREB phosphorylation (pCREB) and increases the expression of BDNF and proBDNF. These neurotrophic factors then act on p75(NTR) and TrkB receptors, facilitating LTP and LTD, ultimately contributing to improved memory performance.

This study found that the activation of brain function after FL ingestion observed in the intervention study was due to the production of BDNF through the activation of memory-related GPCRs (Figure 5). On the other hand, the mechanism by which FL, which is poorly absorbed, phosphorylates CREB in the short period of 30 min after ingestion remains unclear. We hypothesized that this effect is due to the astringency of FL, but the details are unknown. It is imperative to elucidate the true chemical and biological characteristics of the astringency of FL in future studies. Additionally, it is crucial to explore how these short-term effects on brain function influence the enhancement of hippocampus-dependent cognitive function in humans through prolonged FL ingestion.
